# Evaluation of Apoptosis in Multipotent Hematopoietic Cells of Bone Marrow by Anthracycline Antibiotics

**Published:** 2017

**Authors:** Asieh Aramvash, Azra Rabbani Chadegani, Safa Lotfi

**Affiliations:** a *Department of Biochemistry, Institute of Biochemistry and Biophysics, University of Tehran, Tehran, Iran.*; b *Department of Bioscience and Biotechnology, Malek-Ashtar University of Technology, Tehran, Iran.*; c *Department of Biotechnology, Institute of Science and High Technology and Environmental Sciences, Graduate University of Advanced Technology, Kerman, Iran.*

**Keywords:** Apoptosis, Daunorubicin, Doxorubicin, Multipotent hematopoietic cells, PARP

## Abstract

Anthracycline antibiotics are potent anticancer drugs widely used in the treatment of solid tumors and hematological malignancies. Because of their extensive clinical use and their toxic effect on normal cells, in the present study the effect of these drugs on multipotent hematopoietic bone marrow cells was investigated employing, viability tests, PARP cleavage, Hoechst 33258 staining, DNA fragmentation and superoxide anion production techniques. The results revealed that daunorubicin and doxorubicin exhibited time and dose dependent cytotoxicity against the cells and upon increasing the drugs concentrations, apoptosis was occurred after 4 h of incubation and at low concentration of the drugs. The cleavage of poly ADP-ribose polymerase (PARP) demonstrated by daunorubicin and doxorubicin treatment of the cells, suggest that the apoptotic process is PARP dependent. The drugs induced DNA fragmentation and also anion superoxide production was increased upon rising drugs concentrations. From the results it is concluded that anthracycline antibiotics represent cytotoxic effect on hematopoietic progenitor/stem cells of bone marrow, inducing apoptosis and in this process toxicity of daunorubicin is more pronounced compared to doxorubicin.

## Introduction

Bone marrow suppression or myelotoxicity is a serious side effect of chemotherapy . Direct *in-vitro* exposure of bone marrow cells to chemotherapeutic drugs like anthracyclines and alkylating agents has shown a reduction in progenitor survival and stem cell depletion (1, 2). 

Daunorubicin and its congener doxorubicin are two anthracyclines that are widely used in the treatment of solid tumors and hematological malignancies (3). Similar to other anthracyclines, they may also be used to intensify standard conditioning before bone marrow transplantation (4). They mainly act by intercalation of their planar aglycan chromophore between DNA base pairs, and their amino sugar ring lies in the minor groove of the double helix (5, 6). Although chemotherapy affects every organ system in the body, the cell populations that typically exhibit rapid cell turnover, such as those of the bone marrow, are the most sensitive (7). Previous studies on the mechanism of action of both drugs have demonstrated that their cytotoxicity is partially related to two major types of DNA damage namely DNA adducts and protein-associated single and double strand DNA breaks (8). DNA damage can have a variety of biological ramiﬁcations including inhibition of transcription and replication that ultimately leads to cell death (9).

Cell death occur by apoptosis or necrosis depending on the dosage, exposure duration of the drug and cell type involved (10). Recent data have demonstrated that apoptosis may be involved in cell death induced by chemotherapeutic agents such as cisplatin (11), cytarabine (12) and topoisomerase II inhibitors (13-16). 

Thus far, investigations on the action of daunorubicin and doxorubicin have been performed on leukemic cell lines (17). The extensive use of these drugs in clinical oncology and their toxic effect on normal cells such as bone marrow, encouraged us to elucidate the effect of anthracycline anticancer drugs on multipotent hematopoietic cells of bone marrow. In this study cytotoxicity effect of daunorubicin and doxorubicin on the cells was compared and the results suggest that cytotoxicity is mediated by programmed cell death, apoptosis. It is also provided that the effect of daunorubicin is stronger than doxorubicin. 

## Experimental

*Materials*

DMEM with 2 mM L-glutamine (Gibco) was supplemented with 3.7 g/L NaHCO_3_, 10% heat-inactivated fetal calf serum (Faculty of veterinary medicine, University of Tehran) 30 mg/L asparagine, 100 U/mL penicillin and 10 μg/mL streptomycin (Gibco). Anti-PARP (85 kDa fragment) antibody was purchased from Abcam. Trypan blue, MTT, proteinase K, ECoR1-Hind III digested DNA marker, cocktail protease inhibitor, HRP-conjugated IgG, ethidium bromide, cytochrome C, superoxide dismutase were purchased from Sigma-Aldrich chemical company. DPA and other chemical compounds were purchased from Merck. Annexin-V-FLUOS Staining Kit was obtained from Roche. PARP antibody and IgG2a-PE and IgG2b-PE were from AbCam. CD34-PE, C-kit-PE, Sca-1-PE antibodies were a gift from Malek-Ashtar University (Tehran-Iran).

Daunorubicin and doxorubicin were purchased from Helale Ahmar pharmacy (Tehran, Iran, manufactured by Pharmacia) and used without further purification. Stock solutions of the drugs were prepared in sterile distilled water at a concentration of 2 mg/mL and stored at -20 °C in the dark. Before use they were diluted to desired concentrations with PBS, pH 7.4. 

Male balb/c mice weighting 20-25 g (6 to 8 weeks old) were obtained from laboratory animal center of IBB. They were maintained in conventional pathogen free conditions in a temperature (22-23 °C), humidity (50-70%), and photoperiod (12 h dark/light cycle) controlled room. 

*Isolation of multipotent hematopoietic cells of bone marrow and Immunophenotyping*

Total bone marrow from each femur and tibia pair was eluted aseptically with 1mL syringe (25 gauge needle) into 1 mL DMEM and aliquot of the cell suspension was diluted in 3% acetic acid to lyse red blood cells. The total nucleated cells were counted and the viability determined by trypan blue exclusion assay. The bone marrow cells were cultured overnight in DMEM at a density of 10^6^ cells per mL at 37 °C with 5% CO_2_ and fully humidified condition (18). Non-adherent cells which are the population of hematopoietic stem and progenitor cells were collected and used for further investigation.

For immunophenotyping, 50 μL of PBS (pH 7.4) and the following FITC-conjugated monoclonal antibodies, CD34-PE, C-kit-PE, Sca-1-PE, IgG2a-PE and IgG2b-PE, were add­ed to the cells. After 30 min incubation, the cells were rinsed with 2 mL of PBS and finally resuspended in 1% paraform­aldehyde until the time of data acquisition. The data acquisition and analysis were performed by flow cytometry using Becton-Dickinson FACS calibur and WinMDI software.

*Cytotoxicity assay*

Nonadherent multipotent hematopoietic cells of bone marrow (10^6^ cells/mL) were cultured in the absence and presence of various concentrations of daunorubicin or doxorubicin for 4 h (time course study revealed that 4 h is the best incubation time) at 37 °C with 5% CO_2_ and fully humidified condition. Daunorubicin or doxorubicin was added directly at the onset of the culture and the cultures without the drugs were used as control. 

Trypan blue: Drug treated and the controls were subjected to trypan blue (0.4% w/v) exclusion assay and the viability of the cells determined using hemocytometer. 

MTT assay: The method of Mosmann (19) was used with some modifications. The MTT assay is based on the cleavage of tetrazolium salt to form blue formazan dye in viable cells. The cells (8 × 10^4^ cells/well) were seeded into a 96 well plate (Nunclon, Denmark) and incubated with a series of drugs concentrations for 4 h (to control wells, only culture medium was added). Then 10 *μ*L of MTT (5 mg/mL in H_2_O) was added to each well and the cells were incubated at 37 °C with 5% CO_2_ for 4 h. The medium was aspirated and replaced with 100 *μ*L DMSO per well in order to dissolve the formed violet formazan crystals in the viable cells. 

The plates were gently shaken for 5 min at room temperature to allow complete dissolving of the formazan and the absorbance read at 570 nm using a BioTek microplate reader (USA). The percent of cytotoxicity was calculated: % cytotoxicity (cell death) = (1 – [absorbance of experimental wells/absorbance of the control wells]) × 100.

*Fluorescent dye staining*

Hoechst 33258 staining assay was performed, based on the nuclear morphology (20). Multipotent hematopoietic cells were cultured for 4 h in the absence and presence of various concentrations of the drugs as described above. After incubation time, the cells were packed by centrifugation at 1000 × g, washed with PBS and then stained by incubating in 25 μL PBS containing 1 µL of 1 mg/mL stock of Hoechst 33258 at 37 °C for 5 min in the dark. Then the cells were visualized under Zeiss fluorescence microscope with a 40X objective equipped with a digital camera. Apoptotic cells were scored on the basis of nuclear morphology changes, such as chromatin condensation and fragmentation from random fields of view. 

*Gel electrophoresis and immunoblotting of PARP*

After incubation of the cells with various concentrations of daunorubicin or doxorubicin, PARP extraction was performed (21) by resuspending the cells in sample buffer [containing 62.5 mM Tris (pH 6.8), 4 M urea, 10% glycerol, 2% SDS, 5% b-mercaptoethanol and 0.03% bromophenol blue] and the samples were loaded onto a 12% SDS-polyacrylamide gel as described by Laemmle (22). The gel was run for 1.5 h at 100 V, stained with 0.1 % coomassie brilliant blue R 250, destained in 10% methanol/acetic acid and photographed. 

Western blot: The protein samples were run on 12% polyacrylamide gel at 100 V for protein separation. The proteins were then transferred onto a nitrocellulose membrane (23). The membrane with the immobilized protein bands was incubated for 1 h at 37 °C with 1% (w/v) gelatin in Tris-NaCl buffer (50 mM Tris-HCl, pH 7.4, 150 mM NaCl), referred to as blocking buffer, and washed three times of 5 min with Tris-NaCl buffer. The membrane was then incubated overnight with overall anti rabbit 85 kDa fragment of PARP at 4 °C. After three times washing the membrane with Tris-NaCl/Tween 20 (0.05%), it was incubated with peroxidase-conjugated goat anti rabbit IgG for 2 h at room temperature. After washing (three times), the membrane was incubated with the substrate solution (1.2 mL of 0.3% 4-chloro-1-naphtol in methanol was mixed with 20 mL Tris-NaCl buffer and 20 mL H_2_O_2_) for 30 min at 37 °C and the reaction stopped by adding distilled water. The membrane was then dried and photographed.

*Quantification of DNA fragmentation*

The multipotent hematopoietic cells treated with the drugs were collected by centrifugation at 2000 × g for 10 min at 4 °C. The cells were lysed in 0.7 mL of ice cold lysis buffer (5 mM Tris-HCl pH 8, 20 mM EDTA, and 0.5% triton X-100) by incubation at 37 °C for 3 h and then centrifuged at 10000 × g for 20 min at 4 °C. The pellets were resuspended in 0.5 mL of TE buffer (10 mM Tris-HCl, pH 8, 1 mM EDTA). To the pellets (P) and the supernatants (S), 0.5 mL of 10% trichloroacetic acid (TCA) was added and incubated at room temperature for 10 min. The samples were centrifuged for 20 min at 10000 × g and the pellets were resuspended in 0.5 mL of 5% TCA, followed by incubation at 100 °C for 20 min. Subsequently, to each sample 160 μL of diphenylamine solution (150 mg DPA in 10 mL glacial acetic acid, 150 µL of sulfuric acid and 50 µL acetaldehyde16 µg/mL) was added and incubated overnight at room temperature (25). Absorbance was measured at 600 nm and the percentage of DNA fragmentation calculated: OD_600_ of the supernatant/ [OD_600_ of the supernatant + OD_600_ of the pellet] × 100.

*Superoxide anion release assay*

The procedure of Mayo and Curnutte was used (24). After incubation of multipotent hematopoietic cells in the absence and presence of various concentrations of daunorubicin or doxorubicin, the cells were centrifuged for 10 min at 1000 × g. 

The packed cells were washed twice with PBS and then to each sample 200 µL cytochrome C (160 µmol/L) and 200 µL phorbol 12-myristate 13-acetate (10^−6^ mol/L) were added. Also to the control 17 µL (1 µg/mL) SOD (60 U) was added and the samples were incubated at 37 °C for 15 min and then centrifuged at 6000 × g for 10 min at 4 °C. Superoxide anion production was determined from absorbance reading against the control at 550 nm using UV 260 Shimadzu spectrophotometer.

**Figure 1:**
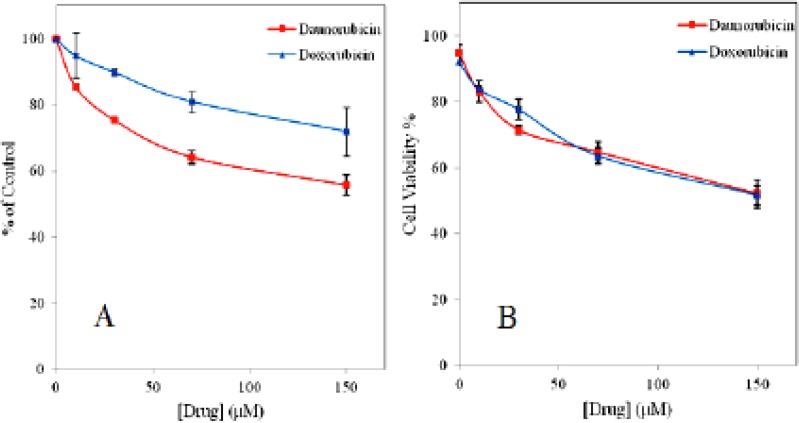
Dose-dependent cytotoxicity of non-adherent multipotent hematopoietic cells treated with various concentrations of daunorubicin and doxorubicin. Cell viability was assessed by trypan blue exclusion (A) and MTT (B) assay. Data are expressed as means ± SD of three independent experiments

**Figure 2:**
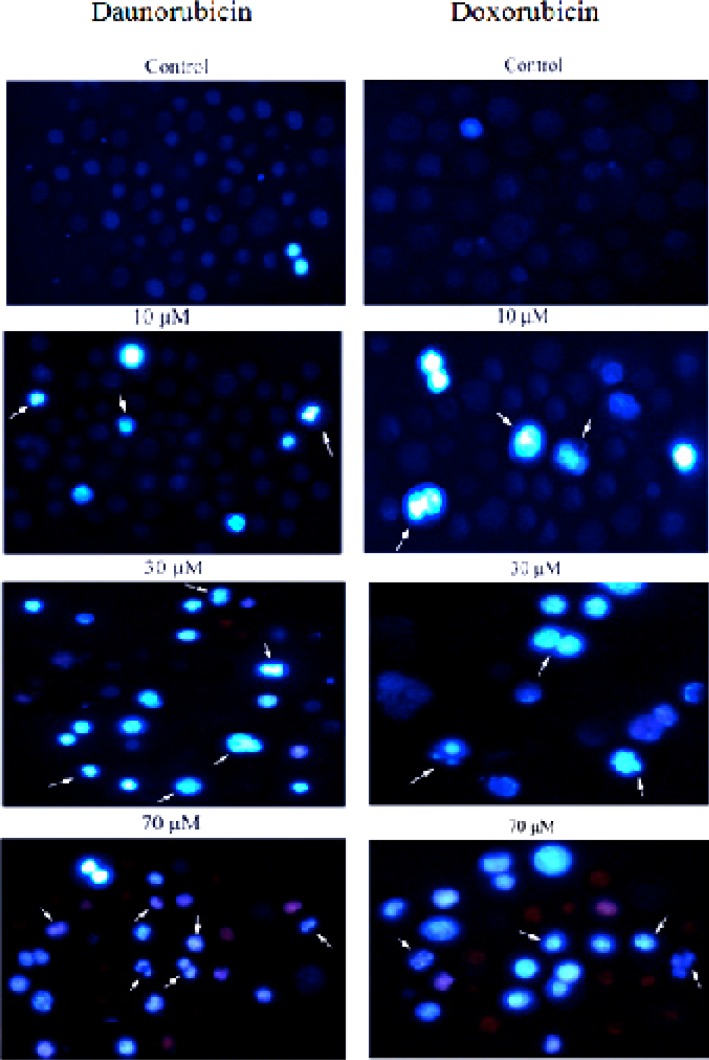
Morphological changes in the non-adherent multipotent hematopoietic cells of mouse bone marrow cells induced in the presence of various concentrations of daunorubicin and Doxorubicin for 4 h and staining with Hoechst 33258. Arrows indicate the cells with condensed chromatin and/or fragmented nuclei. For clarity, only some examples are labeled. (Magnification 200X

**Figure 3:**
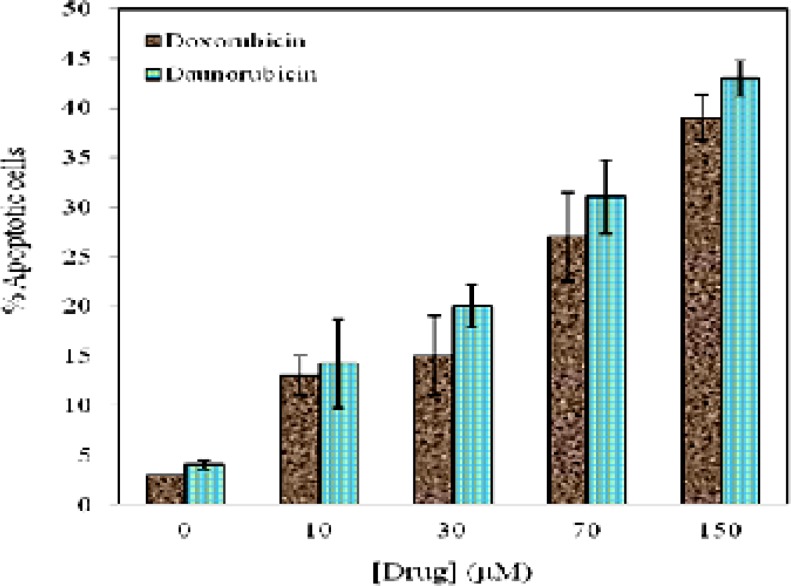
Relative percentage of apoptosis scored on the basis of nuclear morphology changes, such as chromatin condensation and DNA fragmentation from random fields of view for various concentrations of daunorubicin (■) and doxorubicin (■). Data are means ± SD of 3 experiments

**Figure 4:**
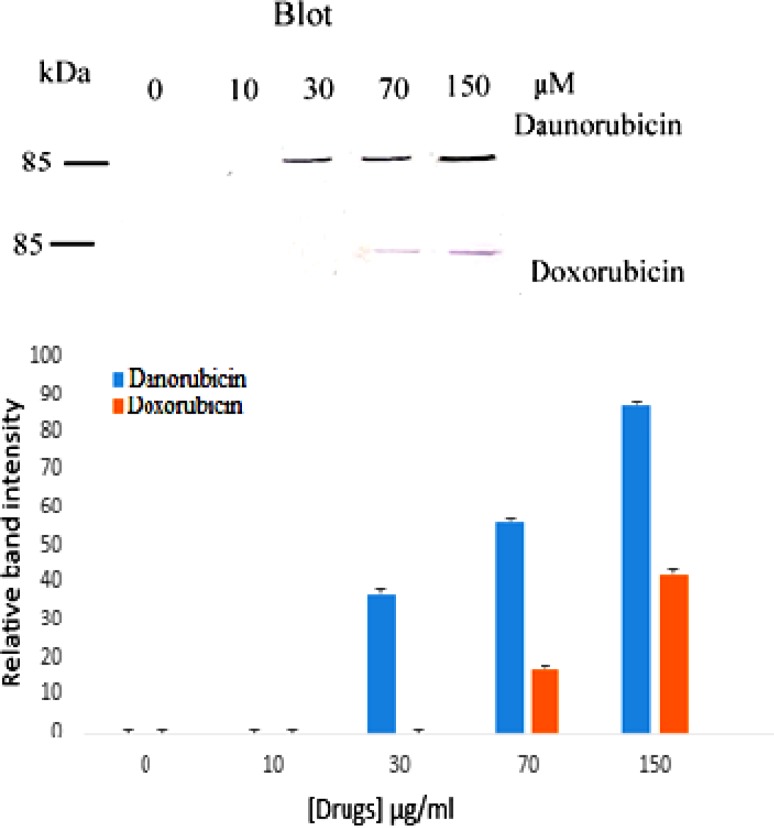
Western blot of PARP cleavage in multipotent hematopoietic cells of bone marrow cells incubated in the absence (Lane 0) and presence of various concentrations of daunorubicin (top) and doxorubicin (bottom) for 4 h. Also the relative band intensity was estimated by Image J is shown. (n = 3).

**Figure 5:**
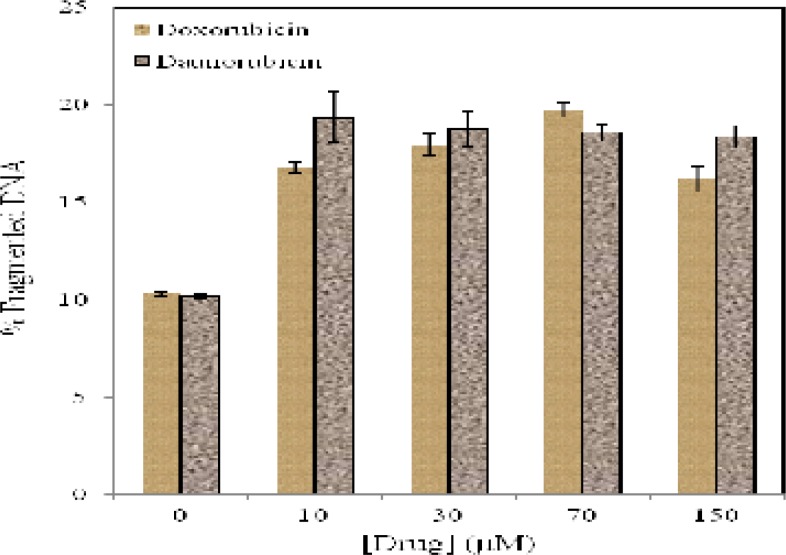
Quantitative estimation of DNA fragmentation in multipotent hematopoietic cells of bone marrow incubated for 4 h in the presence and absence of daunorubicin (■) and doxorubicin (■). Data are means ± SD of 3 experiments (P < 0.05

**Figure 6:**
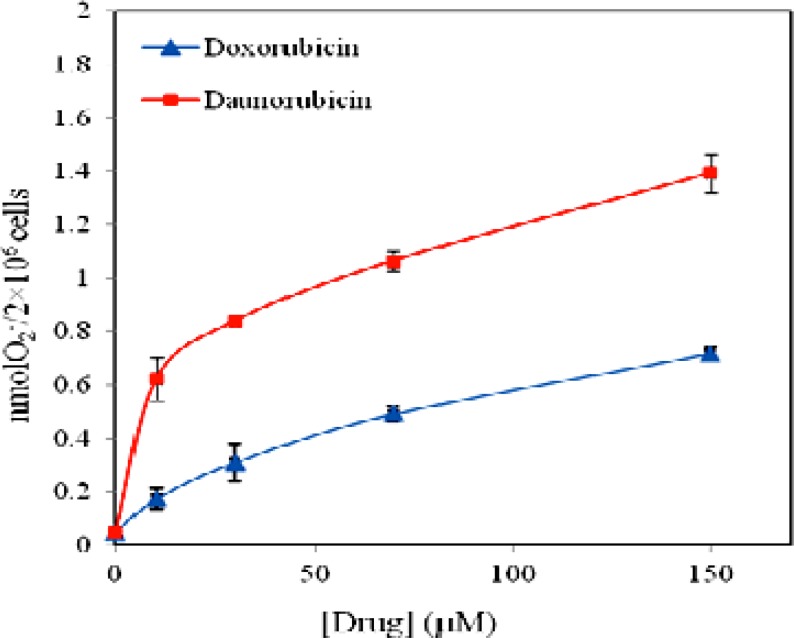
Effect of daunorubicin (■) and doxorubicin (▲) on superoxide anion release from multipotent hematopoietic cells of mouse bone marrow incubated for 4 h. Data are means ± SD of 3 experiments

## Results

The non-adherent bone marrow cells are population rich in hematopoietic progenitor/stem cells as analyzed by Sca-1, CD34, and c-kit expression. The result revealed that more than 90% of the cells were c-Kit^+^, Sca 1^+^ whereas CD34 expression was barely detected (data are not shown). The result is in agreement with the reports indicating that CD34-negative or CD34-low, c-Kit-positive, Sca-1-positive populations are enriched in hematopoietic progenitor cells which have the potency of proliferation and are not fully differentiated (26). We called them multipotent hematopoietic cells of mouse bone marrow.

*Toxicity of daunorubicin and doxorubicin on multipotent hematopoietic cells of bone marrow*

To investigate the potential effect of daunorubicin and doxorubicin on survival rate of the multipotent hematopoietic cells, both trypan blue exclusion and MTT assays were used for comparison. Figure 1 represents dose response curve for both drugs. In trypan blue assay the cells in the absence of the drugs show more than 90% viability up to 4 h of incubation (*e.g.* 92.3%, 95% viability for daunorubicin and doxorubicin, respectively). Whereas the viability is gradually decreased as drugs concentration is increased and even very small amount of drugs show toxicity on these cells. In MTT assay about 45% decrease in the cell viability is obtained for daunorubicin and 30% for doxorubicin when 150 μM of the drugs is used. Although, doxorubicin is more potent drug with strong side effects than daunomycin (27) but it shows less toxicity on these cells. 

The IC50 values of 100 μM and 150 μM was obtained for daunorubicin and doxorubicin, respectively.

*Morphologic changes characteristic of apoptosis in drugs treated cells*

To obtain further insights in the underlying cell death mechanisms, we examined the morphology of the cells upon treatment with various concentrations of daunorubicin and doxorubicin using Hoechst 33258. In this procedure, viable cells display dark blue nuclear fluorescence and apoptotic cells exhibit a bright blue nucleus due to DNA binding capacity of Hoechst 33258 with condensed and fragmented nuclei (28). As is seen in Figure 2, the control shows even distribution of the stain and round homogeneous nuclei feature. Apoptotic cells is gradually increased in a dose-dependent manner and displayed typical changes including reduction of cellular volume, condensed nuclei and stain bright (see arrows).

Figure 3 represents the quantitative analysis obtained from Figure 2. The percentage of apoptotic cells were calculated from different fields of view for both drugs by counting the number of total and apoptotic cells with characteristic of nuclear condensation, cytoplasmic rounding, and membrane blebbing (29). As is seen in Figure 3 a decrease in cell viability is associated with increasing the percentage of apoptotic cells. Within 4 h treatment of the cells with 10 μM of daunorubicin and doxorubicin, 14.2 % and 13% apoptosis is distinguished respectively.

*Effect of the drugs on PARP cleavage*

Poly ADP-ribose Polymerase (PARP) is a nuclear protein which is cleaved to 85 kDa and 25 kDa fragments by caspases during apoptosis (30) and today is a marker of apoptosis. Because apoptosis of different cell types are extremely diverse, we also examined the induction of apoptosis via investigating the cleavage of PARP protein to find whether drug induced apoptosis is PARP related. Figure 4 shows the result. As is seen, no PARP cleavage is detected in the control and at low concentration of both drugs but upon increasing daunorubicin concentration a band at 85 kDa fragments of PARP is observed. Whereas in doxorubicin treated cells PARP cleavage is seen at 70 μM.

*Effect of drugs on DNA fragmentation and superoxide anion production*

Although DNA fragmentation is not the cause of cell death, it seems to be an important feature of apoptosis. Quantitative DNA fragmentation analysis of multipotent hematopoietic cells in the absence and presence of various concentrations of daunorubicin and doxorubicin reveals that (Figure 5) low concentration of daunorubicin (10 µM) produce 19% of DNA fragmentation. In the case of doxorubicin, 19.75 % DNA fragmentation is observed after exposure to 150 µM of the drug.

Reactive oxygen species (ROS) have been implicated in apoptosis induction by a variety of anticancer agents (31). To examine the hypothesis that free radical formation may play role in daunorubicin and doxorubicin toxicity on these cells, we investigated SOD-inhibitable cytochrome c reduction in the medium by assessment of O_2_^- ^production in multipotent hematopoietic cells at various drugs concentrations. Figure 6 represents the results and reveals that both drugs stimulate superoxide anion production. O_2_^- ^production in the controls is 0.04 nmole per 2 × 10^6^ cells but the level of superoxide anion reaches to statistically significant level at 150 μM of both drugs (P < 0.01). Thus, superoxide anion production of 20% and 17% is obtained for daunorubicin and doxorubicin when 150 μM of the drugs is used. In the presence of daunorubicin superoxide anion production is increased and levels off at 30 μM, whereas in the case of doxorubicin, the increment in O_2_^- ^content is less affected, however it also levels off at 30 μM.

## Discussion

Induction of apoptosis in cancerous cells is one of the mechanisms of anticancer effect of anthracyclines, but this effect is also damage normal cells/tissue of body as a side effects. In the present study we attempted to clarify the mechanism by which the death of bone marrow progenitor/stem cells is occurred by two potent anticancer drugs, daunorubicin and doxorubicin, to elucidate the possible side effects of chemotherapy and toxicity of the drugs on these cells.

Treatment of multipotent hematopoietic cells with daunorubicin and doxorubicin results in significant decrease in cell viability and induction of apoptosis in a concentration dependent manner and there is precise correlation between MTT and trypan blue assays. Both drugs are able to decrease the MTT-reducing activity of the cells, although daunorubicin resembles to be more toxic than doxorubicin. Previous studies have shown that anthracyclines are rapidly cleared from plasma and taken up into tissues and persist for several days and cause dose dependent apoptotic cell death in tumor cells (6, 32). In the present experiment it is evident that toxicity occurs even at low concentration of both drugs suggesting that doxorubicin and daunorubicin in apart from affecting tumor cells have significant damaging effects on normal bone marrow stem cells and lead these cells into death even at low concentrations and short period of exposure. 

Hoechst staining of the cells treated with both daunorubicin and doxorubicin display a typical apoptotic form of nuclei that includes chromatin condensation and DNA fragmentation, however, the nuclei of untreated cells stain homogenously. Quantitative analysis of the percent of apoptotic cells confirm and clearly demonstrate susceptibility of the cells to these drugs thus percent of apoptosis is increased upon rising drugs concentration.

Activation of caspase-3 was assessed by the cleavage of one of its substrates, PARP, a 116 kDa protein, into its 85 kDa signature fragment. As stated, both daunorubicin and doxorubicin induce PARP cleavage, consistent with apoptosis and activation of proteases. This finding is in agreement to the finding of Zhang *et al.* (33) showing that daunorubicin strongly induces PARP dependent apoptosis in K562 leukemia cells. Daunorubicin induced cell death has also been reported in muscle stem cells (34). Moreover, it has also been proposed that intercalation of daunorubicin and doxorubicin into cellular DNA results in accumulation of DNA strand breaks (35). The DNA strands break was evaluated by quantitative analysis of fragmented DNA. The results showed an increase in the proportion of fragmented DNA against the negative control in doxorubicin and daunorubicin suggesting induction of apoptosis in multipotent hematopoietic cells. Also in this concept daunomycin represents higher potency than doxorubicin.

ROS, which are byproducts of normal cellular oxidative processes, have been suggested as a regulator of the process involved in the initiation of apoptotic signaling. Under experimental condition used, both drugs increase anion superoxide production in a dose dependent manner. The result is in consistent with previous evidence suggesting that generation of superoxide anion is induced in response to a variety of stimuli like anthracyclines and cause apoptotic cell death (36, 37). The result also confirms that the level of superoxide anion production in daunorubicin treated cells is in higher than when doxorubicin is used. 

From the results presented above it is concluded that anthracycline antibiotics, daunorubicin and doxorubicin, are cytotoxic for normal cells such as multipotent hematopoietic stem cell. In these cells however daunorubicin is more efficient or represents higher toxicity than doxorubicin in inducing cell death and apoptosis. Therefore, usage of anthracyclines in chemotherapy especially in the case of bone marrow transplantation in which immunosuppression is done must be down with caution.
